# Hypertension, diabetes and obesity are associated with lower cognitive performance in community-dwelling elderly: Data from the FIBRA study

**DOI:** 10.1590/1980-57642016dn11-040009

**Published:** 2017

**Authors:** Monalisa Fernanda Bocchi de Oliveira, Mônica Sanches Yassuda, Ivan Aprahamian, Anita Liberalesso Neri, Maria Elena Guariento

**Affiliations:** 1 Post-Graduate Program in Gerontology – Faculty of Medical Sciences, State University of Campinas – Unicamp, Campinas, SP, Brazil.; 2 School of Arts, Sciences and Humanities, University of São Paulo, São Paulo, Brazil.; 3 Department of Internal Medicine, Faculty of Medicine of Jundiaí, Jundiaí, SP, Brazil.; 4 Division of Geriatrics, Department of Internal Medicine, Faculty of Medicine, University of São Paulo, São Paulo, Brazil.

**Keywords:** cognition, hypertension, diabetes mellitus, obesity, elderly, cognição, hipertensão arterial, diabetes mellitus, obesidade, idosos

## Abstract

**Background:**

Systemic hypertension (SH), diabetes mellitus (DM) and abdominal obesity may negatively impact cognitive performance.

**Objective:**

To evaluate the association between SH, DM and abdominal obesity and cognitive performance among cognitively unimpaired elderly.

**Methods:**

A cross-sectional study of individuals aged 65+ from seven Brazilian cities was conducted. SH and DM diagnoses were self-reported and abdominal circumference was objectively measured. Individuals who scored below the education-adjusted cutoff scores on the Mini-Mental State Examination (MMSE) were excluded.

**Results:**

Among 2,593 elderly, 321 (12.38%) had SH, DM and abdominal obesity concomitantly (Group I) and 421 (16.23%) had none of the three diseases (Group II). Group I had a higher proportion of individuals that were women, aged 70-74 years, illiterate and with lower income. Group I had a higher number of participants with low cognitive performance (28.04% vs. 17.58% in Group II). Variables associated with poor cognitive performance were: female gender (OR: 2.43, *p* < 0.001); and lower education (OR: 0.410, *p* < 0.001). The presence of the three diseases and age were not significant in the education-adjusted model.

**Conclusion:**

There was an association between cognition and the presence of SH, DM and obesity. However, education seems to be decisive in determining cognitive performance in the presence of these three conditions.

## RESUMO.

**Introdução:**

Hipertensão arterial sistêmica (HAS), diabetes mellitus (DM) e obesidade abdominal são doenças crônicas comuns entre os idosos e podem afetar negativamente o desempenho cognitivo.

**Objetivo:**

Avaliar a associação entre HAS, DM e OA sobre o desempenho cognitivo de idosos sem comprometimento cognitivo significativo.

**Métodos:**

Estudo transversal de uma amostra probabilística de indivíduos com 65 anos e mais, de sete cidades brasileiras (estudo FIBRA). Variáveis sociodemográficos, diagnóstico de HAS e DM foram relatados pelos participantes e a circunferência abdominal foi medida pelos pesquisadores. Aplicou-se o Mini Exame do Estado Mental (MEEM), excluindo-se os que pontuaram abaixo da nota de corte ajustada para educação.

**Resultados:**

Na amostra de 2.593 idosos, 321 (12,38%) foram identificados como tendo HAS, DM e OA simultaneamente (Grupo I) e 421 (16,23%) não tinham nenhuma das três (Grupo II). O Grupo I evidenciou proporção maior de mulheres, idosos com 70-74 anos, analfabetos e renda mais baixa. O Grupo I apresentou número maior de participantes com baixo desempenho cognitivo (28,04% vs. 17,58% no grupo II). As variáveis associadas com baixo desempenho cognitivo foram: sexo feminino (razão de chances, OR: 2,43, *p* < 0,001); baixa escolaridade (OR: 0,410, *p* < 0,001). A concomitância das três doenças e a idade não foram significativas no modelo ajustado para escolaridade.

**Conclusão:**

Verificou-se associação entre baixo desempenho cognitivo e a presença das três doenças cardiometabólicas (HAS, DM e obesidade). No entanto, a escolaridade parece ser decisiva na determinação do desempenho cognitivo na presença destas três condições.

## INTRODUCTION

Brazil is currently one of the countries with the fastest growing elderly population in the world. Despite increased longevity, life expectancy free of disability is 59.8 years, or about 12 years less than the overall life expectancy.^1^ Chronic diseases account for nearly 70% of disabilities among the elderly in Brazil.^2^ Systemic hypertension (SH) and type 2 diabetes mellitus (DM) are the most common chronic diseases affecting the elderly. Obesity is increasingly frequent among older adults, and has been linked to major cardiovascular outcomes.^3^ These three conditions are core components of the metabolic syndrome which is regarded as a group of cardiovascular risk factors expected to significantly impact public health costs and planning.^4^

SH is currently considered the main risk factor for cardiovascular disease,^1^ and is also linked to cognitive decline and dementia.^5^ Studies provide clear evidence of the link between DM and cognitive decline, both independently or in combination with other chronic diseases.^6^^-^ 10 Although obesity is still more common among younger groups, it increases the likelihood of developing cardiovascular and cognitive disorders in old age.^11^^-^ 15 In addition, obesity is associated with hyperinsulinemia, increasing the risk of cognitive impairment, hypertension, diabetes mellitus, dyslipidemia, heart disease and adverse cardiovascular events.^9^^,^ 14 SH, DM and obesity are among the cardiovascular risk factors for dementia, and these conditions can be modified by preventive interventions, pharmacological treatment or self-care strategies.^2^ The metabolic syndrome, represented by SH, DM and obesity, has been recently associated with an increased incidence of mild cognitive impairment and progression to dementia. Thus, identifying individuals with metabolic syndrome is a promising approach in early interventions to prevent or slow progression of cognitive impairment.^16^

Sousa et al.^2^ noted that dementia is among the major causes of disability in the elderly and that its prevalence is expected to increase as a result of world population ageing, with high social costs, in agreement with recent epidemiological studies.^17^ Alzheimer's disease (AD) and vascular dementia (VD) are the most prevalent dementia sub-types among the elderly. They share several risk factors and may be considered part of the same continuum rather than distinct diseases.^18^^-^ 21 Cardiovascular risk factors are known to be influenced by sociodemographic variables, such as sex, age, education and income.^2^^,^ 22 However, population-based studies are scarce in developing countries such as Brazil, the most populous country in South America. In addition, the interplay between these variables and cognitive performance in old age is not well understood. Therefore, the objective of the present study was to compare the cognitive performance of cognitively unimpaired community-dwelling elderly with self-reported SH, DM and objectively measured abdominal obesity concomitantly, with age-matched individuals without these conditions. In addition, we investigated whether having SH, DM and abdominal obesity concomitantly was associated with lower cognitive performance and whether this association was affected by sociodemographic variables.

## METHODS

### Participants and procedures.

This cross-sectional analysis is part of the FIBRA (Frailty among Brazilian Elderly) study, a population-based multicenter investigation of the frailty profile of Brazilian elderly. It involved random samples of elderly aged 65 and over living in the community stratified by sex and age, recruited from seven cities across the country: Belém, in Northern Brazil; Parnaíba and Campina Grande, in the Northeast; Campinas, Poços de Caldas and Ermelino Matarazzo, in the Southeast; and Ivoti, in the South. The population census tracts in each city were used to calculate sampling units. For cities with populations over 1 million – Campinas and Belém – 90 census tracts were drawn. For the remaining cities – with less than one million inhabitants - 60 census tracts were drawn, except for Ivoti (in the state of Rio Grande do Sul), in which the entire universe of census tracts was considered.^23^ To guarantee an estimated sampling error of 5%, in larger cities, the sample comprised at least 601 subjects, whereas in smaller cities the sample comprised at least 384 subjects for a 4% sampling error.

A total of 3,476 participants were recruited at home by trained undergraduate and graduate students according to inclusion and exclusion criteria established by Fried et al.^24^ and Ferruci et al.^25^ The subjects participated in a single data collection session lasting between 40 and 120 minutes at community centers located near their home, yielding sociodemographic, anthropometric, clinical, frailty and mental status variables, with mental status assessed using the Mini-Mental State Examination (MMSE).^26^ Participants with MMSE scores below the education-adjusted cutoff point (17 for those who could not read; 22 for those with 1 to 4 years of education; 24 for those with 5 to 8 years; and 26 for those with 9 or more years),^23^ did not proceed to the second stage of data collection. The second stage involved self-reported measures of physical and oral health, lifestyle, access to healthcare services, functional capacity, expectations regarding care, depression, social support and life satisfaction due to higher risk of dementia. A total of 883 participants (25.42%) were excluded due to MMSE scores below cut off values. Of the 2,593 remaining participants without cognitive impairment, 321 had concomitant SH, DM and abdominal obesity and were included as members of Group I, while 421 participants did not suffer from any of these three conditions, namely, Group II. The total sample was 742, as participants with one or with two of these conditions were not included in the analyses. Subjects were grouped into four age categories: 65-69; 70-74; 75-79; and 80 and over, and four education categories: 0, 1 to 4, 5 to 8, and 9 or more years of education. Income was measured as monthly family income in minimum wages.

SH and DM were assessed using a questionnaire comprising nine self-reported dichotomous items about chronic conditions that had been diagnosed by a physician in the previous 12 months. For the evaluation of obesity, abdominal obesity was evaluated because it is considered the most important marker of adipose tissue dysfunction and an indication of the presence of insulin resistance. The measurement of waist circumference was conducted with the person standing up, and it was taken at the midpoint between the anterior superior iliac crest and the lower rib edge. The tape-measure was in millimeter increments and placed at the level of the individual's umbilicus. Men with waist circumference over 102 cm and women with waist circumference over 88 cm were considered as having abdominal obesity. These measures were stratified by gender, according to criteria established by the NCEP – ATPIII.^27^

The MMSE overall scores of the participants were divided into quartiles. It was stipulated that a total score below the cutoff point of the first quartile of the score distribution indicated poor cognitive status. It is important to clarify that even the participants regarded as having lower cognitive performance were above the adopted cut-off scores for dementia.

### Statistical analysis.

Categorical variables were analyzed using descriptive statistics through the Chi-square test and Fisher's exact test. The ordinal data were compared by testing their means using the non-parametric Mann-Whitney test and Kruskal-Wallis test (for three or more groups) and by Tukey's and Dunn's *post-hoc* tests. Univariate and multivariate stepwise logistic regression analyses were conducted, considering poor cognitive status as the criterion variable and sex, age, education and Group (I and II) as independent variables. The significance level for all statistical tests was 5%.

### Ethical issues.

The present study was approved by the Ethics Committee at the School of Medical Sciences, State University of Campinas – UNICAMP (Document number 208/2007). At the beginning of the data collection session, participants were informed about the objectives and procedures of the research study, about their right to refuse to participate at any time, and also that measurements were not invasive, that there were no risks to physical and mental health, and that confidentiality of individual data was guaranteed. All candidates that agreed to participate in the study expressed their informed consent by signing a form. At the end of the data collection session, the elderly participants received general advice on health, based on the gathered information, and were given a handbook with guidelines on health and on how to maintain a healthy lifestyle.

## RESULTS

The average age of participants in the total sample was 72.32 ± 5.56 years and there was no statistical difference in age between Groups I and II (*p* = 0.287). The average years of education was 4.33 ± 4.18 for men and 4.02 ± 4.01 for women, with no statistically significant difference *(p* = 0.286) between sexes. There was a significant difference between Groups I and II in relation to education, family income and MMSE scores (Table 1), with higher values in Group II.

**Table 1:** Baseline characteristics of Groups I and II.

	Group I (n= 321) Mean±SD	Group II (n=421) Mean±SD	**p**
Age	72.08 ± 5.59	72.46 ± 5.62	0.287
Education*	3.62 ± 3.77	4.56 ± 4.27	0.003
Income**	3.73 ± 6.22	4.10 ± 4.50	0.011
MMSE	24.45 ± 3.45	25.18 ± 2.81	0.018

Mann-Whitney test;

* years of formal education;

** number of minimum wages earned; MMSE: Mini-Mental State Examination; SD: standard deviation.

In Group I, there was a higher presence of women and of individuals with no formal education. In Group II, there was a higher number of elderly with nine or more years of education (Table 2).

**Table 2:** Comparison of gender, age and education between Groups I and II.

Variables	Categories	Group I	Group II	*p*
Gender	Male	50 (15.67%)	269 (84.33%)	< 0.001^a^
	Female	271 (64.07%)	152 (35.93%)	
Age	65-69	135 (46.55%)	155 (53.45%)	0.476^b^
	70-74	89 (41.98%)	123 (58.02%)	
	75-79	65 (41.67%)	91 (58.33%)	
	>80	32 (38.10%)	52 (61.90%)	
Education*	0	90 (28.04%)	78 (18.57%)	0.002^c^
	1-4	158 (49.22 %)	211 (50.24%)	
	5-8	47 (14.64 %)	69 (16.43%)	
	>9	26 (8.10 %)	62 (14.76%)	

Chi-square test,

a χ^2^ = 173.50, dF = 1;

b χ^2^ = 2.50, dF = 3;

c χ^2^ = 14.40, dF = 3.

* years of formal education.

The mean MMSE score in the total sample (n = 742) was 24.96 ± 3.10. In all, 164 (22.10%) scored below the 1^st^ quartile on the MMSE, and were thus classified as having low cognitive performance. Among illiterate participants, 121 (72.02%) scored below the first quartile on the MMSE (low cognitive performance). Of the participants with 1-4 years of education, 43 (11.65%) had low cognitive performance. Among the groups with 5-8 years or 9 or more years of education, no individual scored below the first quartile on the MMSE.

Regarding the distribution of participants with low cognitive performance in Groups I and II, by level of education, there was a higher number of individuals with low education and low cognitive performance in Group I (Table 3).

**Table 3:** Comparative analysis of education and level of cognitive performance between Groups I and II.

Education*	Lower cognitive performance (n=164)			Higher cognitive performance (n=577)		*p*
	Group I	Group II		Group I	Group II	
0	72 (80%)	49 (62.82%)		18 (20%)	29 (37.18%)	0.013^a^
1-4	18 (11.39%)	25 (11.85%)		140 (88.61%)	186 (88.15%)	0.893^b^
5-8	0	0		47 (100%)	69 (100%)	
>9	0	0		26 (100%)	62 (100%)	

Chi-square test (χ^2^),

a χ^2^ = 6.12, dF = 1;

b χ^2^ = 0.02, dF=1.

* years of formal education.

On the univariate logistic regression analysis (Table 4), all independent variables were associated with lower MMSE scores, i.e. higher age, belonging to Group I, lower education and being female were associated with higher risk for poor cognitive performance.

**Table 4:** Data obtained from univariate logistic regression analysis related to lower cognitive performance (n=742).

Variables	Categories	**P**	OR	95%CI
Gender	Male (ref.)	---	1.00	---
	Female	0.001	1.85	1.28-2.67
Age (y)	65-69 (ref.)	---	1.00	---
	70-74	0.096	1.47	0.93-2.31
	75-79	0.002	2.08	1.30-3.33
	≥ 80	< 0.001	2.65	1.53-4.61
Presence of SH/DM and abdominal obesity	Group II (ref.)	---	1.00	---
	Group I	< 0.001	1.83	1.29-2.59
Education*	Continuous variable (years)	< 0.001	0.420	0.366-0.483

SH: systemic hypertension; DM: diabetes mellitus; OR: Odds ratio for lower cognitive performance; n = 578 with higher performance status and n = 164 with lower performance status. 95%CI = 95% confidence interval. Ref.: Reference level.

* years of formal education.

On the multivariate analysis (Table 5), among the variables studied, only education and being female were selected as being significantly associated with low cognitive performance. Each year of education decreased the chance of low cognitive performance by 59% and being female increased this chance by 2.43.

**Table 5:** Data obtained from multivariate logistic regression analysis related to lower cognitive performance (n=741).

Selected variables	Categories	*P*	OR	95%CI
Education	Continuous variable (years)	< 0.001	0.410	0.356-0.473
Gender	Male (Ref.)	---	1.00	---
	Female	< 0.001	2.43	1.51-3.89

Stepwise regression for selection of variables; OR: Odds Ratio related to lower cognitive performance; n = 578 with higher performance and n = 164 with lower performance; 95%CI = 95% confidence interval; Ref.: reference level;

* years of formal education.

The multivariate analysis was repeated, excluding education from the model. In this analysis, those who were more likely to have low cognitive performance were older participants, women and individuals with all three chronic diseases (Group I) (Table 6).

**Table 6:** Data obtained from multivariate logistic regression analysis considering gender, age, and the composite of hypertension, diabetes and obesity associated with lower cognitive performance (n = 741) excluding education from the model.

Selected variables	Categories	*P*	OR	95%CI
Presence of SH / DM / abdominal obesity	Group II (Ref.)	---	1.00	---
	Group I	0.029	1.57	1.05-2.35
Age (y)	65-69 (Ref.)	---	1.00	---
	70-74	0.058	1.56	0.99-2.47
	75-79	0.001	2.17	1.35-3.50
	≥80	< 0.001	3.01	1.71-5.29
Gender	Male (Ref.)	---	1.00	---
	Female	0.034	1.58	1.04-2.42

SH; systemic hypertension; DM: diabetes mellitus; Stepwise regression for selection of variables; OR: Odds Ratio related to lower cognitive performance; n = 578 with higher performance and n = 164 with lower performance; 95%CI = 95% confidence interval; Ref.: reference level.

## DISCUSSION

The aim of the present study was to compare the cognitive performance of older individuals from a population-based study, carried out in seven locations in Brazil, who were identified as having SH, DM and obesity concomitantly, against participants who did not have these conditions. An additional aim was to evaluate the association of having the three conditions with lower cognitive performance, controlling for relevant sociodemographic variables. In agreement with a previous Brazilian study,^22^ participants in Group I tended to have lower education and lower cognitive performance. One tentative conclusion from the present study is that in developing countries, having lower education raises the risk for cardiovascular risk factors and cognitive impairment concurrently.

Previous papers^28^^-^ 30 have established a correlation between individual cardiovascular risk factors derived from these chronic conditions, and the development of AD and of VD, which are the most common forms of dementia. A composite risk measure of cardiovascular factors can be depicted through the definition of metabolic syndrome, which is mainly defined by core manifestations of SH, DM and obesity. Previously, several community-based studies associated metabolic syndrome with cognitive decline, mild cognitive impairment, and dementia.^31^^-^ 34 The association of AD with VD can be explained by the development of microangiopathic injury, the formation of neurofibrillary tangles and amyloid plaques, as well as by the evidence of effective therapeutic response with the use of acetylcholinesterase inhibitors in patients diagnosed with VD, a drug typically used in patients with AD.^18^^-^ 21 The presence of self-reported SH and DM and of abdominal obesity associated with lower MMSE scores suggests that elderly subjects diagnosed with the three concomitant diseases should be carefully and repeatedly examined and evaluated for symptoms suggestive of cognitive impairment or of dementia.

The participants included in the present study were physically and mentally healthy, with no signs of functional limitation. These data are in line with a U.S. population study that showed a correlation between metabolic syndrome and mild cognitive impairment.^35^ In contrast, Forti et al.,^36^ besides not having found a correlation between the metabolic syndrome diagnosed in old age and the risk of developing dementia, found that from 75 years of age and over metabolic syndrome was associated with lower risk of developing dementia. In another study, Muller et al.^37^ showed that the metabolic syndrome was not associated with dementia, but that diabetes and hyperinsulinemia were separately associated with metabolic syndrome. On the other hand, Raffaitin et al.^38^ found that, despite the observed association of metabolic syndrome with cognitive decline among French elderly subjects, no correlation was noted between hypertension, abdominal obesity and glucose intolerance, and cognitive impairment. However, the same study found a correlation between DM or SH associated with DM – but not with glucose intolerance – and cognitive decline. Therefore, despite conflicting results, research points to a higher risk of cognitive impairment in the presence of diseases that affect the cardiovascular and metabolic systems, which might be explained by an increase in inflammatory markers and in cell and vascular damage.^39^

The frequency of scores falling within the lower MMSE quartile in the studied sample was 28.04% for Group I, a percentage significantly higher than that of Group II (17.58%). This finding is consistent with data collected by Yaffe et al.^39^ who showed a 26% incidence of cognitive impairment among elderly people with the metabolic syndrome. Therefore, the concomitance of the three above-mentioned conditions – SH, DM and abdominal obesity – seems to negatively affect MMSE scores of cognitively healthy elderly individuals. However, it should be noted that Group I also has lower education and income, compared with Group II.

The higher percentage of women than men suffering from the three concomitant diseases is in line with data from other studies about the metabolic syndrome.^39^ The correlation among older age, being female and having poorer cognitive performance was also reported in earlier studies.^40^^,^ 41

The limitations of the present study are primarily related to its cross-sectional design, which does not allow the establishment of causal relationships. Additionally, since the elderly subjects in the sample were those who scored above the MMSE cutoff point for dementia, it is possible that older individuals, those at more advanced stages of any of the diseases, and participants with less formal education were under represented. Another limitation of the present study is the lack of information on the duration of the diseases in question (SH, DM and abdominal obesity) up to the cognitive assessment date, and whether or not these diseases were treated over the years. The fact that SH and DM status was based on self-report may also be considered a limitation, as participants might have been unaware of these conditions.

Nevertheless, the present paper provides evidence to support early intervention programs regarding modifiable variables that affect the prevention and control of hypertension, diabetes and obesity.^40^ Present results also suggest that improved education levels and disseminating information about chronic disease prevention in old age might help to delay cognitive impairment in developing countries. In conclusion, results indicated an association between cognition and the presence of SH, DM and obesity. However, education seems to be decisive in determining cognitive performance in the presence of these conditions.
